# Excess intraoperative fluid volume administration is associated with pancreatic fistula after pancreaticoduodenectomy: A retrospective multicenter study

**DOI:** 10.1097/MD.0000000000006893

**Published:** 2017-06-02

**Authors:** In Woong Han, Hongbeom Kim, JinSeok Heo, Min Gu Oh, Yoo Shin Choi, Seung Eun Lee, Chang-Sup Lim

**Affiliations:** a Department of Surgery, Dongguk University, College of Medicine, Gyeongju, Gyeongsang Province; b Department of Surgery, Samsung Medical Center, Sungkyunkwan University School of Medicine; c Department of Surgery, Chung-Ang University College of Medicine; d Departments of Surgery, Seoul Metropolitan Government-Seoul National University Boramae Medical Center, Seoul National University College of Medicine, Seoul, Korea.

**Keywords:** intraoperative fluid volume, pancreatic fistula, pancreaticoduodenectomy

## Abstract

Recent studies on perioperative fluid administration in patients undergoing pancreaticoduodenectomy (PD) have suggested that increased fluid loads are associated with worse perioperative outcomes. The purpose of this study was to investigate the relationship between intraoperative fluid (IOF) administration and postoperative pancreatic fistula (POPF), and to determine additional risk factors affecting pancreatic fistula in patients undergoing PD.

From 2005 to 2014, a total of 182 patients with various periampullary diseases after PD were reviewed retrospectively at Dongguk University Ilsan Hospital, Chung-Ang University Hospital, and Dongnam Institute of Radiological and Medical Sciences. Patients were assigned to high or low IOF groups based on more or less fluid administration for supplementation of estimated blood loss and maintenance volume (12.5 mL/kg/h) than planned, respectively. The associations between IOF administration, pancreatic fistula development, and perioperative outcomes were evaluated.

A total of 98 patients were assigned to the high-IOF group, and 84 to the low-IOF group. Risk factors for pancreatic fistula after univariate analysis were assignment to the high-IOF group, higher preoperative serum hemoglobin level, ampullary or bile duct cancer, pylorus preserving PD, small pancreatic duct, duct-to-mucosa pancreatojejunostomy, use of a stent, and mesh application to pancreatojejunal anastomosis. Among these, assignment to the high-IOF group (hazard ratio [HR] = 5.501, 95% CI 1.624–18.632, *P* = .006) and a small (<4 mm) pancreatic duct (HR = 4.129, 95% CI 1.569–14.658, *P* = .035) were identified as independent risk factors for the development of pancreatic fistula after multivariate analysis. However, long-term survival rate did not differ according to IOF group or duct size.

Excessive IOF volume administration is associated with an increased incidence of pancreatic fistula after pancreaticoduodenectomy.

## 1 Introduction

Fluid therapy is a controversial topic in perioperative management.^[1–3]^ The excessive administration of fluid can impair pulmonary, cardiac, and gastrointestinal functions, contributing to postoperative complications and prolonged recovery.^[4]^ On the other hand, surgical patients are more likely to have serious complications and die if they have limited physiological reserves. Adequate fluid administration can reduce the stress response to surgical trauma and can support recovery. There are no standard guidelines for intraoperative fluid (IOF) volume administration with regard to speed of infusion, total volume, or type of fluid. According to textbook recommendations, IOF administration in patients undergoing intraabdominal procedures should be in the range of 10 to 15 mL/kg/h with replacement of blood volume losses with crystalloid solutions at a 3:1 ratio or colloid solutions at a 1:1 ratio.^[5–7]^ This regimen, which favors liberal administration of fluid to prevent hypovolemia and decrease end-organ perfusion, is not, however, evidence-based. In addition, intravenous fluid overload during surgery has been shown to decrease muscular oxygen tension and delay recovery of gastrointestinal function.^[8,9]^ Furthermore, postoperative weight gain and IOF overload are associated with poor survival^[10]^ and complications.^[11,12]^ As a result, defining the optimal strategy for perioperative fluid administration in major abdominal surgery remains a challenging clinical problem.^[13]^ Pancreaticoduodenectomy (PD) or pylorus-preserving PD (PPPD) is the standard surgical procedure for treating benign or malignant diseases of the periampullary region. Despite improvements in the perioperative care of these patients and patient selection for surgery, PD is a high-risk, technically demanding operation associated with substantial perioperative morbidity and mortality.^[14–17]^ Furthermore, despite advancements in operative technique and improvements in postoperative outcomes, postoperative pancreatic fistula (POPF) is a frequent and potentially life-threatening complication following PD that affects nearly one-quarter of patients.^[15,17–19]^ It is the factor most often linked with postoperative mortality, certain complications such as delayed gastric emptying, longer hospital stays, readmissions, and increased costs. Furthermore, it frequently delays timely delivery of adjuvant therapies, and reduces overall patient survival.^[20]^ Several approaches to reduce POPF after PD have been suggested, but no real progress has been made for at least a decade.

The relationship between POPF and excessive IOF is unclear. Excessive IOF could theoretically be a potent risk factor for POPF after PD because it could cause pancreatic parenchymal and intestinal edema, resulting in pancreaticojejunal anastomotic malfunction or disruption. However, there are few reports of clinical experience with perioperative fluid administration during PD,^[1,14]^ and those that exist are based on small populations or are single-center studies. The purpose in this retrospective multicenter study was therefore to investigate the relationship between IOF administration and POPF and to determine additional risk factors that affect POPF in patients undergoing PD.

## 2 Methods

### 2.1 Patients

With approval from the institutional review boards, all patients with periamullary benign or malignant disease who underwent surgical resection at Dongguk University Ilsan Hospital, Chung-Ang University Hospital, or Dongnam Institute of Radiological and Medical Sciences were reviewed retrospectively between 2005 and 2014. All enrolled hospitals are tertiary referral centers in South Korea.

### 2.2 Definition of intraoperative fluid administration group

**Figure 1:**
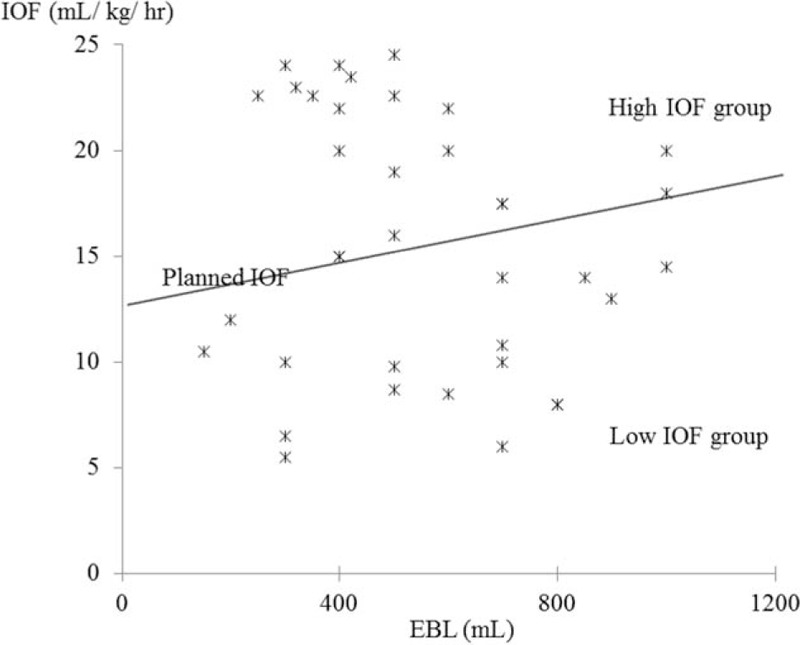
Groups of IOF determined based on EBL and maintenance volume. High-IOF group: actual IOF ≥planned IOF; low IOF group: actual IOF <^∗^planned IOF; planned IOF volume: EBL replacement + maintenance volume EBL replacement amount: 1:1 for colloids and blood components, and 3:1 for crystalloids. Maintenance volume: 12.5 (10–15) mL/kg/h. EBL = estimated blood loss, IOF = intraoperative fluid.

Patients with PD were administrated fluids such as crystalloid solutions, colloid solutions, volume expanders, or blood components intravenously during operation. Planned IOF volume was determined based on the estimated blood loss (EBL) and the maintenance volume. The EBL replacement amount was calculated based on a 1:1 ratio for colloids and blood components and a 3:1 ratio in the case of crystalloids.^[5–7]^ Maintenance volume was set to 12.5 (10–15) mL/kg/h based on previous recommendations (Fig. 1).^[5–7]^ After calculating the planned IOF amount, patients were assigned to high- or low-IOF groups based on whether the actual IOF amount was more or less than the planned IOF (Fig. 1).

### 2.3 Comparison of clinicopathological variables including POPF in patients with PD

Clinical variables including age, gender, body mass index (BMI), American Society of Anesthesiologists (ASA) class, combined comorbidities, and duration of postoperative hospital stay were evaluated. Operative details and histological findings, such as primary diseases, type of surgery, duration of surgery, size of tumor, diameter of pancreatic duct, and methods of pancreatojejunostomy were also evaluated and described in accordance with the 7th edition TNM staging system of the American Joint Committee on Cancer.^[21]^ POPF was defined according to the International Study Group of Pancreatic Fistulas (ISGPF) ^[22]^ as amylase-rich fluid that is, an amylase concentration of more than 3 times the serum concentration collected from the drain placed intraoperatively on day 3 or by needle aspiration for intra-abdominal collection. Other complications such as postoperative hemorrhage, delayed gastric emptying, biliary fistula, intra-abdominal fluid collection, wound infection, and 30-days mortality were also evaluated. Patients were followed-up regularly in outpatient clinics every 3 to 6 months, and information was obtained during follow-up for all patients. Overall survival was analyzed from the date of surgical resection to the date of death from all causes. Causes of death were determined from medical records. The follow-up period was defined as the interval between the date of surgical resection and that of the last follow-up. All data for this study were assessed by dedicated study nurses who entered data in a specifically designed computerized database (MDB, Seoul, Korea).

### 2.4 Statistical analysis

Data were analyzed using Statistical Package for the Social Sciences version 21.0 (SPSS, Chicago, IL). Continuous and normally distributed variables are presented as medians and ranges. Continuous parameters in each group were compared using the independent *t*-test or Mann–Whitney *U*-test, while categorical parameters were compared using the *χ*^2^ test or Fisher's exact test. Medical records and survival data were obtained for all patients. Survival curves were constructed using the Kaplan–Meier method and differences in survival were evaluated using the log-rank test. Multivariate analysis to determine risk factors associated with developing pancreatic fistula was based on Cox's proportional hazards model. Probability (*P*) values of .05 or less were considered statistically significant.

## 3 Results

### 3.1 Clinical characteristics of enrolled patients

Clinicopathological findings in patients with PD according to IOF group are listed in Table 1. Most clinical variables were similar between low- and high-IOF groups except BMI (24.5 ± 3.5 vs 22.5 ± 2.8, *P* <.001), preoperative bilirubin level (1.7 ± 1.8 v. 2.6 ± 2.7, *P* = .010), and follow-up duration (33.0 vs 23.7 months, *P* = .036).

**Table 1:**
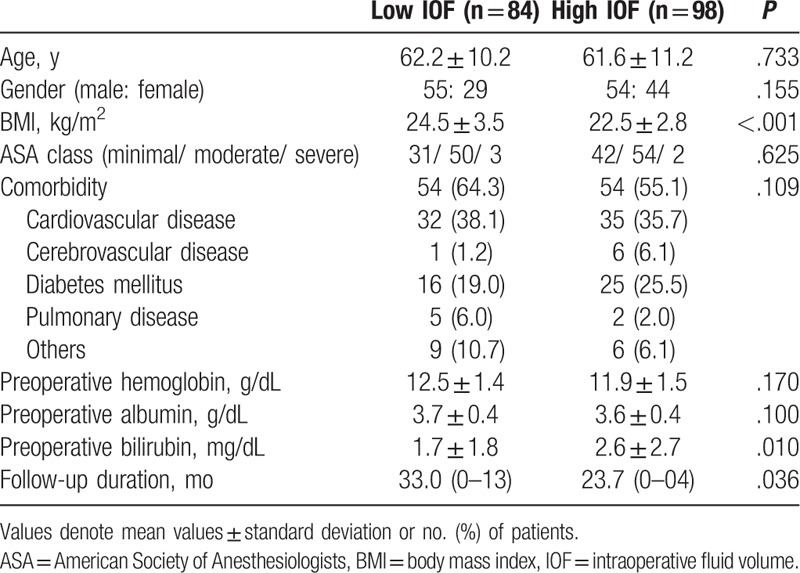
Clinical characteristics of patients enrolled in each group.

### 3.2 Operative details and pathologic data

Operative details and pathologic data for patients with PD according to IOF group are presented in Table 2. Most of these variables were similar between the 2 IOF groups (Table 2).

**Table 2:**
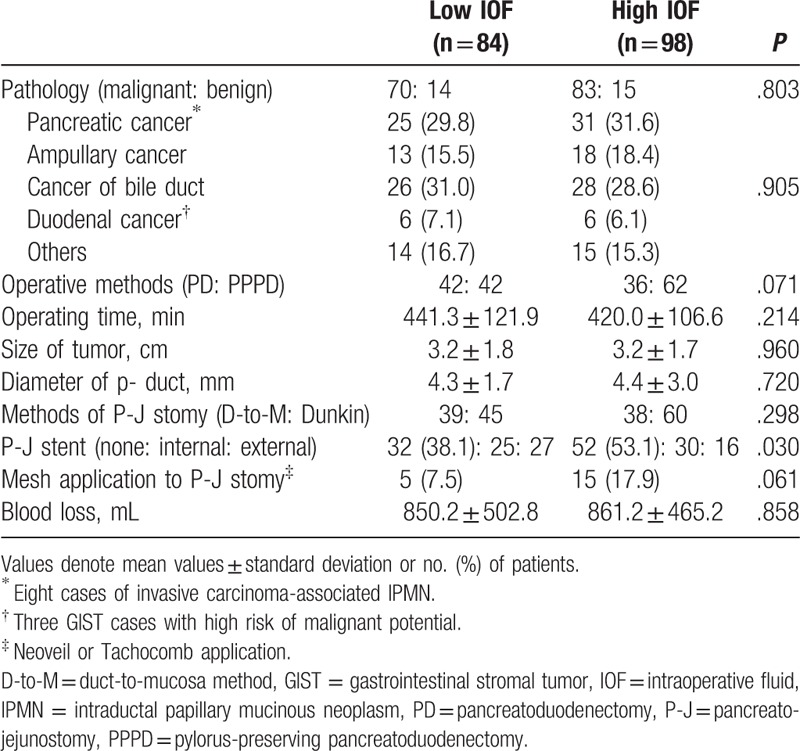
Operative details and pathologic data in the 2 IOF groups.

### 3.3 Comparison of postoperative complications between the 2 IOF groups

The incidence of POPF in patients in the low-IOF group (n = 13, 15.5%) was lower than that in the high-IOP group (n = 27, 27.6%) with statistical significance (*P* = .048; Table 3). However, other complications were not significantly different between the high- and low-IOF groups (Table 3).

**Table 3:**
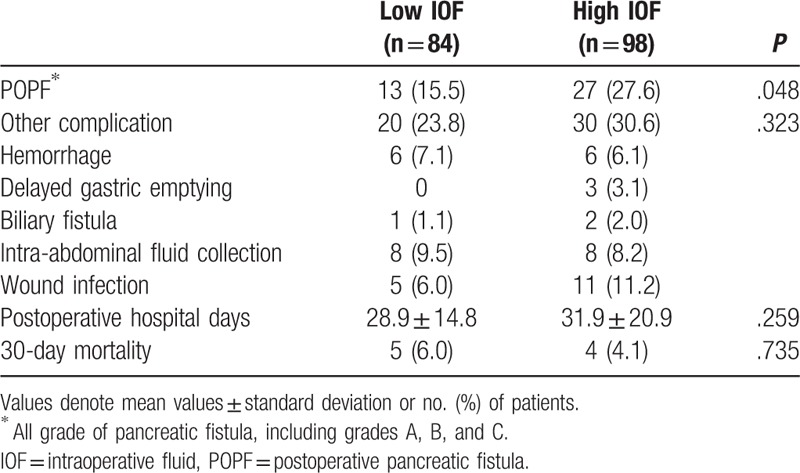
Comparison of postoperative complications between the 2 IOF groups.

### 3.4 Risk factor analysis and survival of POPF in patients with PD/PPPD

**Figure 2:**
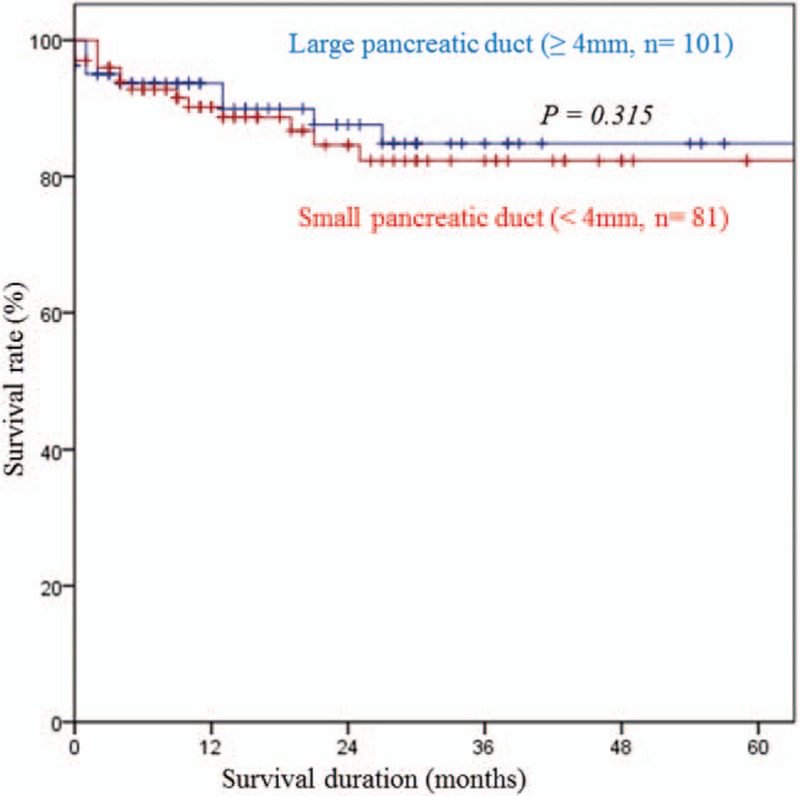
Survival curves according to pancreatic duct size.

**Figure 3:**
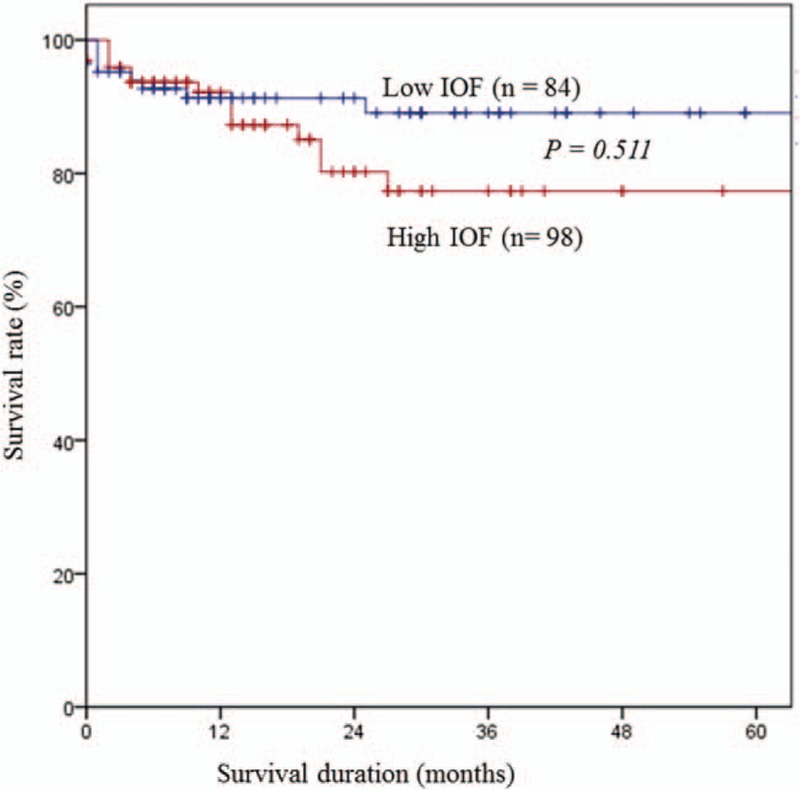
Survival curve of high and low IOF groups. IOF = intraoperative fluid.

Risk factors for pancreatic fistula in univariate analysis were assignment to the high-IOF group, higher level of preoperative serum hemoglobin level, ampullary or bile duct cancer, PPPD, small pancreatic duct, duct-to-mucosa pancreatojejunostomy, use of a stent, and mesh application to pancreatojejunal anastomosis (Table 4). Among these, assignment to the high-IOF group (hazard ratio [HR] = 5.501, 95% CI 1.624–18.632, *P* = .006) and a small (<4 mm) pancreatic duct (HR = 4.129, 95% CI 1.569–14.658, *P* = .035) were identified as independent risk factors for POPF after multivariate analysis (Table 5). However, survival rate did not differ according to the IOF group (Fig. 2) or pancreatic duct size (Fig. 3).

**Table 4:**
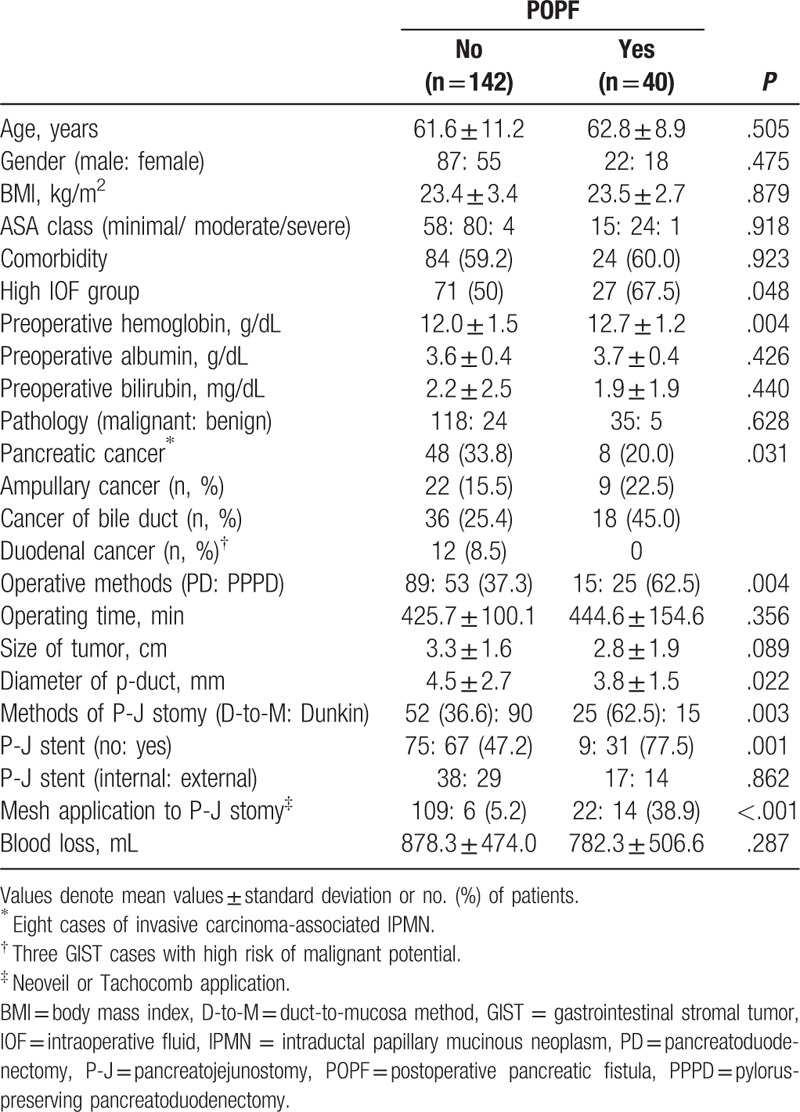
Risk factor analysis for POPF in patients with PD.

**Table 5:**
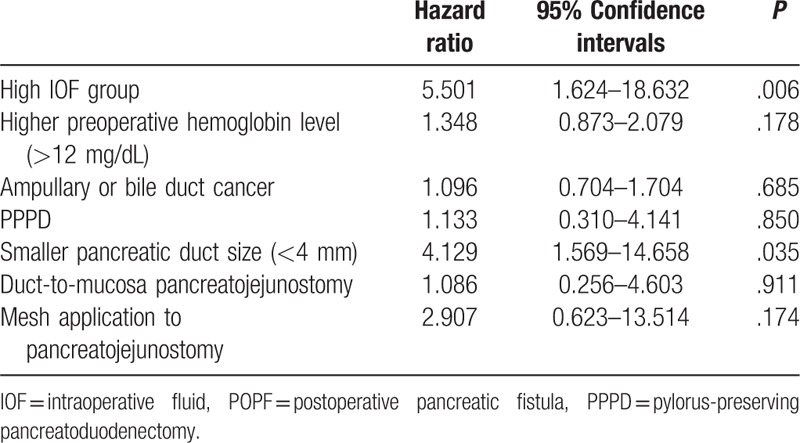
Multivariate analysis of risk factors for POPF.

## 4 Discussion

**Figure 4:**
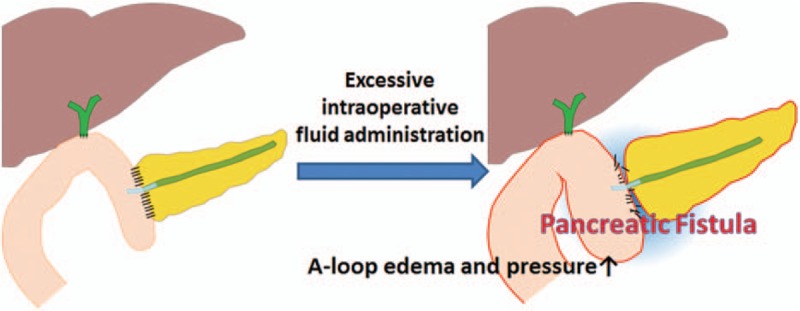
The mechanism of IOF overload that may cause POPF. A-loop indicates afferent loop. IOF = intraoperative fluid, POPF = postoperative pancreatic fistula.

Perioperative fluid therapy has received increased interest in recent years because several studies have demonstrated that the strategy used for fluid therapy can influence the postoperative outcome.^[4,23]^ Perioperative fluid administration in major abdominal surgery, particularly gastrointestinal surgery, is undergoing a fundamental change, as previous dogma supporting liberal fluid administration practices has been challenged.^[13]^ Several studies have suggested that relative IOF restriction in patients who are undergoing intraabdominal surgery reduces the number of patients who experience complications and shortens the time to recovery of gastrointestinal function and to hospital discharge.^[9,24,25]^ Particularly, recent studies on perioperative fluid administration in patients undergoing PD have suggested that increased fluid loads are associated with worse perioperative outcomes.^[1,13,14,26]^ Moreover, Wang et al^[14]^ reported that high-IOF administration is associated with an increased incidence of POPF after PD. In that study, the patients were categorized according to volume of IOF. POPF rates were significantly greater in the high-IOF group (≥8.2 mL/kg/h) (*P* = .035). Fischer et al^[26]^ observed that not only the frequency, but also the severity of complications related to pancreatic anastomosis were greater in the IOF group relative to the low-IOF group. Acute normovolemic hemodilution group was administrated more IOF than the standard group. Severe complications (more than grade 3) were more common in high-IOF group (32% vs 23.1%, *P* = .17) and pancreatic anastomosis-related complications also were significantly higher in the high-IOF group (21.5% vs 7.7%, *P* = .045). In our study, membership of the high-IOF group was identified as an independent risk factor for POPF after multivariate analysis (Table 5). We therefore believe that high IOF is associated with an increased incidence of POPF after PD. Despite advancements in operative techniques and improvements in postoperative patient care, POPF is widely considered to be the most common major complication, with a reported incidence of 10% to 40%.^[17,20,22,27–29]^ Patients who develop a POPF require more invasive interventions, remain hospitalized longer, and incur greater hospital costs as points accumulate.^[15,20]^ As a result, many attempts have been made to reduce the incidence of POPF by technical variations of reconstruction after PD, use of somatostatin analogs, pancreatic stenting, and pancreatic drainage among other approaches.^[17]^ The mechanism by which the overload of intravenous fluids causes POPF is poorly understood. However, there is some evidence that fluid overload diminishes tissue oxygenation and leads to impaired gut motility as well as mucosal edema.^[14,30]^ Fluid overload may also result in deficient pancreatojejunostomy healing and excessive IOF can cause intraoperative metabolic acidosis,^[29,31]^ which could be a risk factor for developing POPF.^[29]^ As a result, it is our belief that high IOF causes ischemia and poor healing of the pancreatic anastomosis by tissue edema from aggressive volume replacement in a “rebound” fashion. Resultant swelling of the anastomosis can result in duct occlusion or suture disruption due to increased afferent loop pressure (Fig. 4). Based on analyses of pre- and intraoperative variables, excessive intraoperative blood loss has been shown to be a significant risk factor for POPF in some studies,^[20,28,32]^ whereas it was not significant in other studies.^[29,33,34]^ In our study, blood loss itself was not an independent risk factor (Table 4). The volume of fluid used to replace the EBL appears to be more important than EBL itself for developing POPF in terms of tissue edema and to further challenge the integrity of the pancreatic-enteric anastomosis (Tables 4 and 5).

In this study, although excessive IOF administration was identified as an independent risk factor for POPF, there was no significant difference between the low and high fluid volume groups in terms of survival rate (Fig. 2). This may be because there was no significant difference between the groups in the rates of clinically relevant POPF such as ISGPF grade B or C pancreatic fistulas (data not shown). More observational or prospective studies are needed to confirm the effect of IOF volume on clinically relevant POPF development and survival in patients with PD. Several studies have suggested that a small pancreatic duct might predict a patient's risk of developing a POPF.^[18,20,35]^ A narrowed pancreatic duct diameter is not only more challenging to reconstruct, but also more likely to either occlude or dehisce. A smaller duct accommodates fewer sutures than a larger one, and does not facilitate juxtaposition of the duct to bowel mucosa as easily.^[20]^ In this study, we provided additional evidence that a pancreatic duct smaller than 4 mm is an independent risk factor for developing POPF based on multivariate analysis (Table 5). The present study considered most risk factors for POPF based on clinical, operative, and pathologic variables reported in the published literature. Older age, male gender, high BMI, high ASA score, presence of comorbidities, and surgery due to malignancy have previously been defined as patient-dependent preoperative risk factors for morbidity and mortality following pancreatic surgery.^[22,28,32,35,36]^ However, in our study, none of the aforementioned factors had a relationship with an increase in risk of POPF. Needless to say, pre- or intraoperative risk assessment is more desirable than postoperative quantification of POPF risk.^[15]^ Recently, there has been a paradigm shift among pancreatic surgeons in the management of POPF, from a reactive “wait and see” approach that depends on treating fistulas when they become evident, to a proactive strategy that instead relies on early anticipation and timely prevention through attempted prophylaxis.^[20,37]^ However, although there are many risk factors that affect POPF that can be assessed postoperatively, such as histological features, gland characteristics, or drain amylase level,^[19,20]^ these are variables that are uncontrollable or uncorrectable. For this reason, reducing the IOF volume might be a simple way to reduce POPF after PD. The present study did have some limitations. First, this study was retrospective and observational in nature, so it is possible that not all details in the patients’ records were recorded correctly. As such, the outcomes might reflect selection bias that could not be measured. Furthermore, the possibility that the observed differences were due to factors other than the amount of resuscitation fluid cannot be excluded due to the retrospective nature of this study. Another potential limitation of this study is that we selected all patients with POPF (ISGPF grade A, B, or C) and not just those with ‘clinically relevant’ POPF (grades B and C). In this study, all variables studied were compared between patients with all grades of POPF and those with either grade B or C, but no significant difference was found (data not shown). This may in part relate to the small numbers of patients with grades B or C POPF.

In conclusion, excessive IOF administration is associated with an increased incidence of POPF after PD. Adequate IOF administration may considerably reduce the rate of POPF after PD. A better understanding of the effects of fluid administration is necessary to decrease the rate of occurrence of POPF after PD. Further clinical investigation and randomized studies are needed to determine whether this relationship is causal or not.
